# Sodium Nitroprusside-Induced Activation of Vascular Smooth Muscle BK Channels Is Mediated by PKG Rather Than by a Direct Interaction with NO

**DOI:** 10.3390/ijms23052798

**Published:** 2022-03-03

**Authors:** Hristo Gagov, Irina V. Gribkova, Vladimir N. Serebryakov, Rudolf Schubert

**Affiliations:** 1 Department of Animal and Human Physiology, Faculty of Biology, St. Kliment Ohridski University of Sofia, 1164 Sofia, Bulgaria; hgagov@uni-sofia.bg; 2 Scientific and Clinical Department, State Budgetary Institution «Research Institute for Healthcare Organization and Medical Management of Moscow Healthcare Department», 115088 Moscow, Russia; igribkova@yandex.ru; 3 Institute of Experimental Cardiology, Cardiology Research Center, 121552 Moscow, Russia; 4 Physiology, Institute of Theoretical Medicine, Faculty of Medicine, University of Augsburg, 86159 Augsburg, Germany

**Keywords:** arteries, smooth muscle, calcium activated potassium channel, nitric oxide, sodium nitroprusside, protein kinase G

## Abstract

Nitric oxide (NO) is a powerful vasodilator in different vascular beds and NO-donors are widely used in clinical practice. Early data suggested that NO and NO-donors activate vascular smooth muscle high-conductance, calcium-activated potassium channels (BK channels). There exist two hypotheses explaining the effect of NO and NO-donors on BK channels—one stating that protein kinase G (PKG) mediates the effect of NO, and the other one stating that NO acts directly on the channel. Thus, the degree of contribution of PKG to the NO-induced activation of the BK channel is still not completely clear. This study tested the hypothesis that the sodium nitroprusside (SNP)-induced activation of vascular smooth muscle BK channels is fully mediated by PKG. This hypothesis was investigated using the patch-clamp technique and freshly isolated smooth muscle cells from rat tail artery. In whole-cell experiments, SNP considerably increased the outward current compared with the addition of the bath solution. SNP did not alter the current in the presence of iberiotoxin, the specific blocker of BK channels, during co-application with hydroxocobalamin, an NO-scavenger, and in the presence of Rp-8-Br-PET-cGMPS, the specific PKG-inhibitor. In inside-out patches, the activity of BK channels was increased by SNP, SNAP, and DEA-NO. However, these effects did not differ from the effect of the application of drug-free bath solution. Furthermore, a similar increase in single BK channel activity was induced by Rp-8-Br-PET-cGMPS, Rp-8-Br-PET-cGMPS together with SNP, hydroxocobalamin, and hydroxocobalamin together with SNP or DEA-NO. Finally, the activity of excised BK channels did not change in the absence of any application but was considerably increased by PKG compared with the addition of drug-free bath solution. These results suggest that NO released from NO-donors stimulates the BK current only through activation of PKG.

## 1. Introduction

Blood flow to different organs is regulated by changes in artery diameter. Factors released from the endothelium are among the most potent regulators of vessel diameter. Nitric oxide (NO) is a powerful endothelium-derived vasodilator in different vascular beds. For this reason, NO-donors are widely used in clinical practice.

It has been shown that the vasodilating effect of NO is mediated by several mechanisms. Early data suggested that NO and NO-donors activate vascular smooth muscle high-conductance, calcium-activated potassium channels (BK channels), e.g., [1,2,3]. Activation of BK channels is thought to produce hyperpolarization of vascular smooth muscle cells, leading to closure of voltage-dependent calcium channels, a subsequent decrease in intracellular calcium concentration, and vasodilation.

Recently, we have demonstrated that NO-donors evoke the deactivation of BK channels at intermediate and low levels of vessel contractility [4]. This effect, particularly that induced by the NO-donor sodium nitroprusside (SNP), was suggested to be due to an NO-induced reduction in calcium influx via l-type calcium channels. Thus, BK channels have been shown to be able to limit NO-induced vasodilation. In contrast, we have also shown that activation of BK channels facilitates NO-induced vasodilation at higher levels of contractility. Of note, the latter effect induced by SNP was only partially blocked by Rp-8-Br-PET-cGMPS, a selective protein kinase G (PKG) inhibitor [5]. This partial block might be due to incomplete inhibition of PKG by Rp-8-Br-PET-cGMPS at the concentration used [4]. Unfortunately, higher concentrations of Rp-8-Br-PET-cGMPS could not be employed because, at such concentrations, this substance may stimulate basal PKG activity [6,7]. Thus, our data obtained on intact vessel preparations could not clarify the extent to which PKG contributes to the SNP-induced activation of the BK channel.

Indeed, there exist two hypotheses explaining the effect of NO and NO-donors on vascular smooth muscle BK channels. One states that these agents activate soluble guanylate cyclase, resulting in an increased formation of cGMP and subsequent activation of PKG. PKG then increases the activity of BK channels. This has been shown in excised membrane patches or isolated smooth muscle cells from, for example, porcine coronary artery [8], rabbit cerebral artery [2], human pulmonary artery [9], mouse aorta [10], and rat cerebral artery [11] smooth muscle cells as well as for cloned smooth muscle BK channels [12]. The other hypothesis claims that NO activates BK channels independently of PKG, and a direct action of NO on the channel has been suggested. Thus, BK channels were activated in excised membrane patches after application of NO to the intracellular side of the channel in rabbit aorta [13] and of NO and the NO-donors SIN-1 and SNP in rat mesenteric artery [14] smooth muscle cells. Hence, even when taking into account the data of our recent study together with previously published results, we were unable to clarify the degree of contribution of PKG to the SNP-induced activation of the BK channel.

Therefore, this study tested the hypothesis that the SNP-induced activation of vascular smooth muscle BK channels is fully mediated by PKG. In order to obtain direct evidence, BK channel activity was measured in excised membrane patches and BK channel currents were recorded in freshly isolated vascular smooth muscle cells.

## 2. Results

### 2.1. Effect of SNP on the Outward Current of Rat Tail Artery Smooth Muscle Cells

In whole-cell experiments on rat tail artery smooth muscle cells, an outward current was found consisting mainly of a BK-current (for details see [15]).

**Figure 1:**
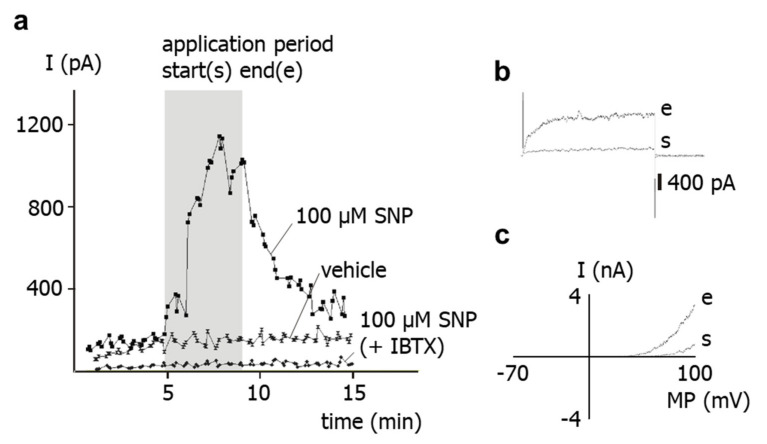
**Effect of the NO-donor SNP on the outward current of rat tail artery smooth muscle cells.** (**a**) Examples of the time course of the effect of SNP, SNP in the presence of 300 nM iberiotoxin (IBTX), and experimental bath solution (vehicle) on the outward current. The application period is shown as grey area; start of application is marked as (s), end of application as (e). The current was elicited with use of a 500 ms-long voltage step from a holding potential of −40 mV to a test potential of +70 mV. (**b**) Examples of traces of the outward current elicited with use of a 500 ms-long voltage step from a holding potential of −40 mV to a test potential of +70 mV at the start (s) and at the end (e) of the application of SNP. (**c**) Examples of current–voltage (I–MP) relationships of the outward current elicited with use of a 1.5 s-long voltage ramp from a holding potential of −40 mV in the voltage range from −70 to +100 mV at the start (s) and at the end (e) of the application of SNP.

Application of the NO-donor SNP at 100 µM to intact cells increased the outward current reversibly. The concentration of 100 µM was selected because preliminary experiments have shown that this is the lowest concentration producing maximum activation of the current. In the example shown in Figure 1a,b, the outward current increased from 163 pA to 1009 pA. The increase in outward current was observed at different potentials (Figure 1c). In contrast, application of experimental bath solution (vehicle) to intact cells did not alter the outward current in time control experiments (Figure 1a). Further, in the presence of 300 nM iberiotoxin, the specific blocker of BK channels [16,17], application of SNP at 100 µM to intact cells did not change the outward current (Figure 1a).

**Figure 2:**
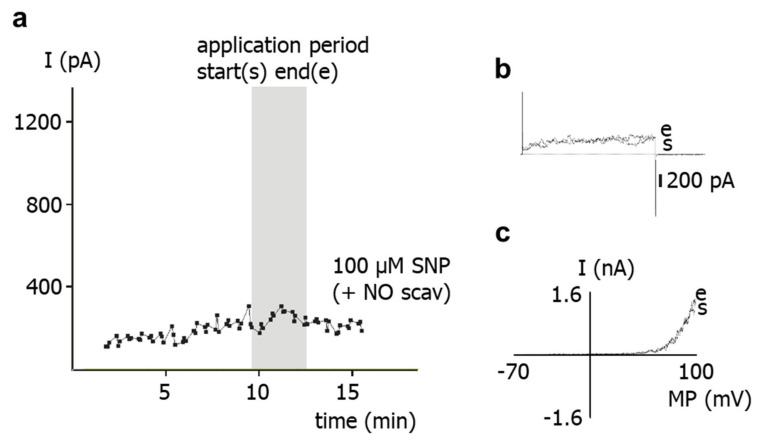
**Effect of the NO-donor SNP on the outward current of rat tail artery smooth muscle cells in the presence of the NO-scavenger hydroxocobalamin.** (**a**) Example of the time course of the effect of SNP in the presence of 1 mM hydroxocobalamin (NO scav) on the outward current. The application period is shown as grey area; start of application is marked as (s), end of application as (e). The current was elicited with use of a 500 ms-long voltage step from a holding potential of −40 mV to a test potential of +70 mV. (**b**) Examples of traces of the outward current elicited with use of a 500 ms-long voltage step from a holding potential of −40 mV to a test potential of +70 mV at the start (s) and at the end (e) of the application of SNP in the presence of 1 mM hydroxocobalamin. (**c**) Examples of current–voltage (I–MP) relationships of outward current elicited with use of a 1.5 s-long voltage ramp from a holding potential of −40 mV in the voltage range from −70 to +100 mV at the start (s) and at the end (e) of the application of SNP in the presence of 1 mM hydroxocobalamin.

To study the role of NO released from SNP for the effect of the latter on the outward current, the NO-scavenger hydroxocobalamin [18] was used. Thus, the experimental bath solution was supplemented with 1 mM hydroxocobalamin. In the continuous presence of hydroxocobalamin, application of 100 µM SNP to intact cells did not alter the outward current (Figure 2).

**Figure 3:**
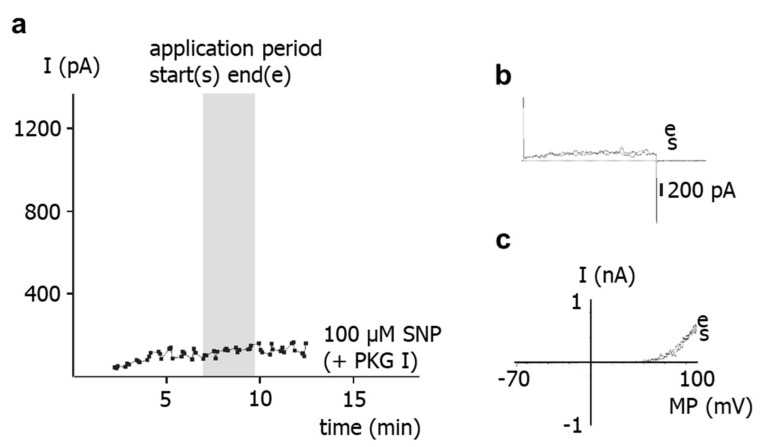
**Effect of the NO-donor SNP on the outward current of rat tail artery smooth muscle cells in the presence of the PKG-inhibitor Rp-8-Br-PET-cGMPS.** (**a**) Example of the time course of the effect of SNP in the presence of 1 µM Rp-8-Br-PET-cGMPS (PKG I) on the outward current. The application period is shown as grey area; start of application is marked as (s), end of application as (e). The current was elicited with use of a 500 ms-long voltage step from a holding potential of −40 mV to a test potential of +70 mV. (**b**) Examples of traces of the outward current elicited with use of a 500 ms-long voltage step from a holding potential of −40 mV to a test potential of +70 mV at the start (s) and at the end (e) of the application of SNP in the presence of 1 µM Rp-8-Br-PET-cGMPS. (**c**) Examples of current–voltage (I–MP) relationships of outward current elicited with use of a 1.5 s-long voltage ramp from a holding potential of −40 mV in the voltage range from −70 to +100 mV at the start (s) and at the end (e) of the application of SNP in the presence of 1 µM Rp-8-Br-PET-cGMPS.

To study the role of PKG for the effect of SNP on the outward current, the PKG-inhibitor Rp-8-Br-PET-cGMPS [5] was used. Thus, the experimental bath solution was supplemented with 1 µM Rp-8-Br-PET-cGMPS. In the continuous presence of Rp-8-Br-PET-cGMPS, application of 100 µM SNP to intact cells did not alter the outward current (Figure 3).

**Figure 4:**
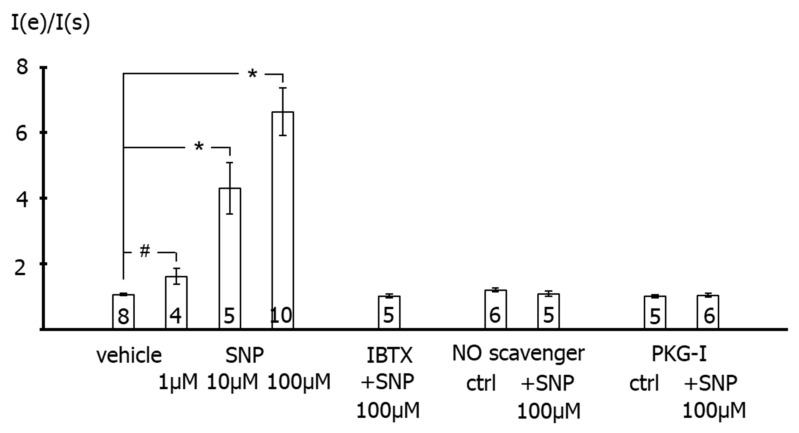
**Effect of the NO-donor SNP on the outward current of rat tail artery smooth muscle cells.** Data are expressed as the ratio of the current at the end of the application period under specified experimental conditions (I(e)), i.e., application of experimental bath solution in the time control series (vehicle), SNP, SNP in the presence of 300 nM iberiotoxin (IBTX), 1 mM hydroxocobalamin alone (NO scavenger ctrl) and together with SNP, and 1 µM Rp-8-Br-PET-cGMPS alone (PKG-I ctrl) and together with SNP, to the current at the beginning of the application period under the same conditions (I(s)). Only the effects of SNP alone were different from vehicle control; number of cells investigated appears on bar for each condition. #—*p* < 0.05. *—*p* < 0.001.

On average, the outward current was increased by 1, 10 and 100 µM SNP at +70 mV compared to vehicle application (*p* < 0.05, *p* < 0.001, *p* < 0.001, respectively) (Figure 4, left part). In contrast, in the presence of 300 nM iberiotoxin, we did not detect any effect of 100 µM SNP on the outward current compared with vehicle application (*p* = 0.52) (Figure 4, left part). Further, we did not detect any effect of 100 µM SNP on the outward current in the presence of 1 mM of the NO-scavenger hydroxocobalamin compared to the application of hydroxocobalamin alone (*p* = 0.22) (Figure 4, middle part). Finally, we did not detect any effect of 100 µM SNP on the outward current in the presence of 1 µM of the PKG-inhibitor Rp-8-Br-PET-cGMPS compared to the application of Rp-8-Br-PET-cGMPS alone (*p* = 0.66) (Figure 4, right part).

### 2.2. Effect of SNP on Single BK Channel Activity

In inside-out patches from rat tail artery smooth muscle cells, the predominantly observed channel showed a conductance of about 170 pS, a steep voltage-dependence, and a remarkable dependence of channel activity on the intracellular free calcium ion concentration. In outside-out patches, 1 mM TEA and 100 nM iberiotoxin blocked this channel [15,19]. All these properties are characteristic of the vascular smooth muscle BK channel.

**Figure 5:**
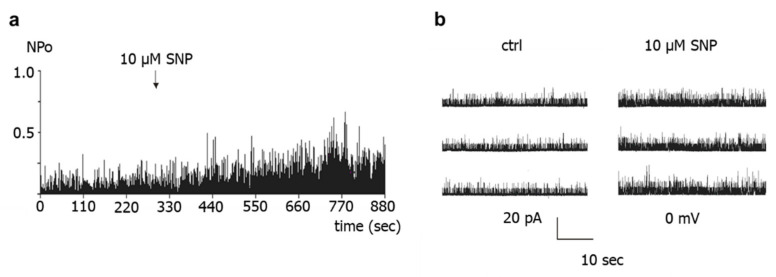
**Effect of the NO-donor SNP on excised BK channels of rat tail artery smooth muscle cells.** (**a**) Example of a diary plot of BK channel activity (NPo) at 0 mV with application of SNP (at arrow). (**b**) Examples of traces of BK channel activity at 0 mV taken from the same patch as in (**a**) during control (ctrl) and addition of SNP.

Application of 10 µM and of 100 µM of the NO-donor SNP to the intracellular side of BK channels increased channel activity. The concentrations of 10 µM and 100 µM were selected because they are in the concentration range used in other studies, demonstrating an effect of NO or NO-donors on excised BK channels. In the example shown in Figure 5, channel activity NPo increased from 0.146 to 0.328 at 0 mV, i.e., 2.25-fold after addition of 10 µM SNP.

**Figure 6:**
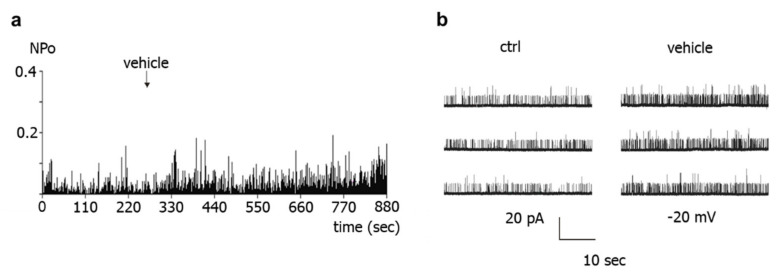
**Effect of experimental bath solution (vehicle) on excised BK channels of rat tail artery smooth muscle cells.** (**a**) Example of a diary plot of BK channel activity (NPo) at −20 mV with application of vehicle (at arrow). (**b**) Examples of traces of BK channel activity at −20 mV taken from the same patch as in (**a**) during control (ctrl) and addition of vehicle.

Further, application of experimental bath solution (vehicle) to the intracellular side of BK channels also activated the channels. This is shown in the example in Figure 6, where NPo increased from 0.038 to 0.066 at −20 mV, i.e., 1.74-fold. 

**Figure 7:**
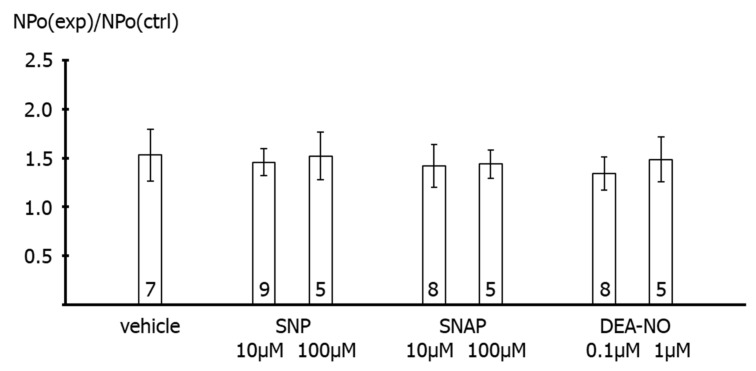
**Effect of NO-donors on excised BK channels of rat tail artery smooth muscle cells.** Data are expressed as the ratio of BK channel activity (NPo) at the end of the application period under specified experimental conditions (NPo(exp)), i.e., application of experimental bath solution in the time control series (vehicle), SNP, SNAP and DEA-NO, to BK channel activity before the beginning of the application period under the same conditions (NPo(ctrl)); number of cells investigated appears on bar for each condition.

On average, we did not detect any effect of 10 µM SNP (*p* = 0.99) or 100 µM SNP (*p* = 0.99) as well as of other NO-donors, 10 µM SNAP (*p* = 0.96), 100 µM SNAP (*p* = 0.88), 0.1 µM DEA-NO (*p* = 0.52), and 1 µM DEA-NO (*p* = 0.93) on the activity of excised BK channels compared to the application of vehicle in the time control series (Figure 7). Single channel amplitude was not altered in these experiments. 

**Figure 8:**
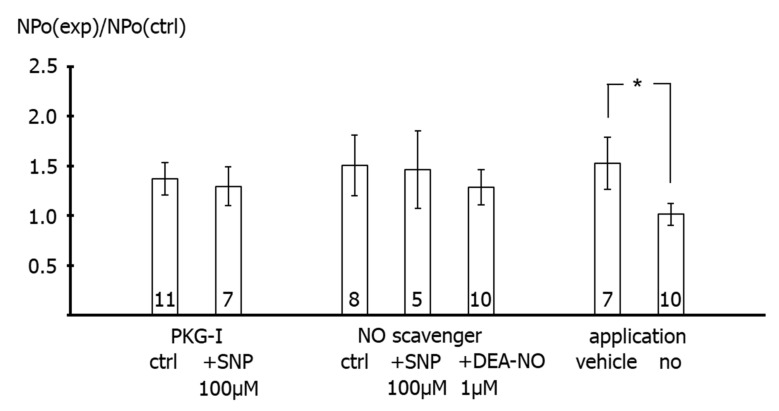
**Effect of NO-donors on excised BK channels of rat tail artery smooth muscle cells.** Data are expressed as the ratio of BK channel activity (NPo) at the end of the application period under specified experimental conditions (NPo(exp)), i.e., application of 1 µM Rp-8-Br-PET-cGMPS alone (PKG-I ctrl) and together with SNP, 1 mM hydroxocobalamin alone (NO scavenger ctrl) and together with SNP or DEA-NO, experimental bath solution in the time control series (vehicle) and no application at all, to BK channel activity before the beginning of the application period under the same conditions (NPo(ctrl)); number of cells investigated appears on bar for each condition. *—*p* < 0.05.

Furthermore, we did not detect any effect of 100 µM SNP on the activity of excised BK channels in the presence of 1 µM Rp-8-Br-PET-cGMPS compared to the application of Rp-8-Br-PET-cGMPS alone (*p* = 0.60) and of 1 µM Rp-8-Br-PET-cGMPS alone compared to vehicle application (*p* = 0.86) (Figure 8, left part). Additionally, we did not detect any effect of 100 µM SNP and of 1 µM DEA-NO on the activity of excised BK channels in the presence of 1 mM hydroxocobalamin compared to the application of hydroxocobalamin alone (*p* = 0.83 and *p* = 0.90, respectively) and of 1 mM hydroxocobalamin alone compared to vehicle application (*p* = 0.87) (Figure 8, middle part). Finally, the activity of excised BK channels was higher during vehicle application than in the absence of vehicle application (*p* < 0.05) (Figure 8, right part). Single channel amplitude was not altered in these experiments.

**Figure 9:**
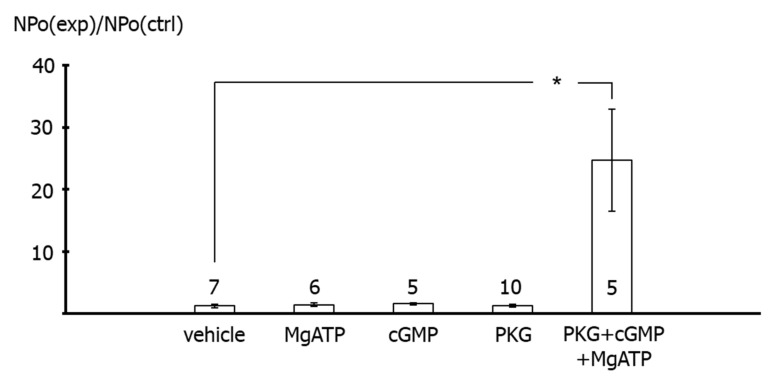
**Effect of PKG on excised BK channels of rat tail artery smooth muscle cells.** Data are expressed as the ratio of BK channel activity (NPo) at the end of the application period under specified experimental conditions (NPo(exp)), i.e., application of 100 µM MgATP, 100 µM cGMP, 6 U/µL PKG, PKG together with MgATP and cGMP, and experimental bath solution in the time control series (vehicle), to BK channel activity before the beginning of the application period under the same conditions (NPo(ctrl)); number of cells investigated appears near the bar for each condition. *—*p* < 0.05.

In addition, we did not detect any effect of 100 µM MgATP (*p* = 1.0), 100 µM cGMP (*p* = 1.0) or 6 U/µL of the catalytic subunit of PKG (*p* = 1.0) on the activity of excised BK channels compared to vehicle application (Figure 9). In contrast, the activity of excised BK channels was higher during the joint application of 100 µM MgATP, 100 µM cGMP and 6 U/µL of the catalytic subunit of PKG compared with vehicle application (*p* < 0.05) (Figure 9). Single channel amplitude was not altered in these experiments.

## 3. Discussion

The results of the present study show that application of the NO-donor SNP to freshly isolated rat tail artery smooth muscle cells increases their outward current considerably. Previously, it has been shown that the outward current of these cells consists mainly of an iberiotoxin-sensitive current, i.e., represents a BK current [15]. This finding suggests that the SNP-induced increase in the outward current observed in the present study is due to a stimulation of the BK current by SNP. Indeed, the present study also demonstrated that SNP was unable to affect the residual outward current after pre-treatment of the cells with iberiotoxin, i.e., under conditions where BK channels are blocked. Therefore, it is concluded that the increase in the outward current of rat tail artery smooth muscle cells after SNP application is due to an SNP-induced stimulation of a BK current.

SNP is a well-known NO-donor. The SNP-induced stimulation of the BK current observed in the present study is most likely due to NO. Indeed, we demonstrated that application of SNP did not increase the BK current in the presence of the NO-scavenger hydroxocobalamin [18]. Furthermore, hydroxocobalamin itself had no effect on the BK current and on excised BK channels. In addition, as mentioned in methods, hydroxocobalamin removed NO from solutions. Consequently, an interaction between NO and hydroxocobalamin may explain the lack of effect of SNP on the BK current in the presence of hydroxocobalamin. Importantly, strong support for this notion was provided in our recent study on intact rat tail arteries, i.e., the vessel from which the cells investigated in the present study were isolated [4]. In the previous study, the vasodilatory effect of SNP was abolished by hydroxocobalamin and was not mediated by either cyanide, which could also be released by SNP [20], or nitroxyl (HNO), which may be generated by the direct interaction of H_2_S with SNP [21]. Thus, data from excised BK channels, freshly isolated single smooth muscle cells and intact vessel preparations from rat tail artery consistently demonstrate that the stimulation of the BK current after SNP application is solely due to NO released from SNP.

In contrast to the coherent data on the contribution of NO in mediating the effect of SNP on BK currents in rat tail arteries, the role of PKG, the canonical mediator of NO-induced effects [22,23], is still unclear. Thus, in our recent study on intact rat tail arteries, the vasodilatory effect of SNP was only partially blocked by Rp-8-Br-PET-cGMPS, a selective PKG inhibitor [4,5]. Of note, it has been suggested that the effects of NO and NO-donors could be explained by a direct action of NO on the BK channel [13,14,24]. However, a direct, PKG-independent effect of NO on rat tail artery BK channels seems unlikely based on the following findings of the present study.

First, application of the NO-donors SNP, SNAP and DEA-NO to the intracellular side of excised BK channels from rat tail artery smooth muscle cells increased the activity of the channel by about 50–60%, independent of the NO-donor concentration and the NO release mechanism of the respective NO-donor. A similar elevation of the activity of excised BK channels has been reported for NO, SIN-1 and SNP in rat mesenteric arteries [14] and for NO in rabbit aorta [13]. In contrast, no change in channel activity was observed with the NO-donors SNP and SNAP on the BK channel from rat mesenteric arteries [25] and on the cloned BK channel from human aorta [26]. Importantly, extending the findings of previous reports, we observed in the present study that the increase in BK channel activity was not different from the increase in single channel activity observed after application of the solvent of the NO-donors in time control experiments. Moreover, additional experiments on excised BK channels designed to interact with either NO released from the NO-donors or with PKG, which mediates the effect of NO on BK channels, all produced a similar effect. Thus, the increase in single BK channel activity induced by Rp-8-Br-PET-cGMPS did not differ from the effect of Rp-8-Br-PET-cGMPS together with SNP, the effect of hydroxocobalamin did not differ from the effect of hydroxocobalamin together with SNP as well as with DEA-NO, and the effects of Rp-8-Br-PET-cGMPS and hydroxocobalamin did not differ from the effect of the solvent alone.

In contrast, application of the catalytic subunit of PKG together with MgATP and cGMP to the intracellular side of excised BK channels from rat tail artery smooth muscle cells increased the activity of the channel considerably. This effect of PKG most likely indicates phosphorylation of the BK channel by PKG, because MgATP, cGMP and the catalytic subunit of PKG alone were not able to affect BK channel activity. The combined effect of MgATP, cGMP and the catalytic subunit of PKG was required to observe an increase in BK channel activity. Of note, these findings are consistent with numerous similar observations, e.g., [2] and reviewed in [27]. Importantly, using the same method, we had previously shown that excised BK channels from rat tail artery smooth muscle cells can be activated by application of protein kinase A (PKA) [19] and inhibited by application of protein kinase C (PKC) [15]. Thus, the data shown in the present study, as well as previously published data, provide strong evidence that excised BK channels can be activated under the experimental conditions used in the present study.

Further, the present study shows that BK channel activity was higher during vehicle application to excised BK channels than in the absence of such application. This effect cannot be explained by an alteration in calcium concentration, because the calcium concentration was the same on both sides of the patch and was buffered with EGTA. However, similar changes in BK channel activity have been reported previously during longer recordings and referred to as Wanderlust kinetics [28]. Thus, mechanical stimulation of the BK channel due to the application maneuver or application-related changes in the microenvironment of the channel or slow fluctuations in open probability with time, which underlie Wanderlust kinetics, could explain the increase in the activity of excised BK channels during vehicle application. Of note, the search for the reason of this phenomenon was not the focus of the present study. Regardless of the cause of this phenomenon, the occurrence of similar alterations in channel activity after application of different substances, substance combinations, and of the experimental bath solution indicates that the observed effect is not due to a specific direct interaction with the BK channel, in particular not to an interaction induced by NO. In conclusion, we did not obtain any evidence that the NO-donor-induced activation of excised BK channels observed in our study could be due to a direct action of NO on the BK channel. 

Second, in freshly isolated single smooth muscle cells, application of SNP did not affect the BK current at all in the presence of the PKG-inhibitor Rp-8-Br-PET-cGMPS. It was also shown that Rp-8-Br-PET-cGMPS itself had no effect on the BK current and on excised BK channels. As mentioned in the methods, Rp-8-Br-PET-cGMPS produced only a small, if any, reduction in NO concentration, which cannot explain the lack of effect of SNP in the presence of Rp-8-Br-PET-cGMPS. Thus, since, in the presence of Rp-8-Br-PET-cGMPS, NO is still present and the channel is still functioning, the lack of effect of SNP should be caused by an action of Rp-8-Br-PET-cGMPS on components of signal transduction pathways that mediate the effect of NO on the BK channel, most likely PKG [5]. Consistent with this interpretation of our data, a complete inhibition of the effect of NO or NO-donors on vascular smooth muscle BK currents has been reported previously. Thus, BK currents were not affected after application of a PKG-inhibitor and SNP in rat mesenteric arteries [25], of a guanylate cyclase inhibitor and SNP in porcine coronary arteries [29], of a PKG-inhibitor and NO in human pulmonary arteries [9], of a PKG-inhibitor and SNAP in rabbit coronary arteries [30] and of DEA-NO in PKG-knock-out mouse aorta [10]. Therefore, the stimulation of the BK current after application of SNP observed in our study is most likely mediated only by PKG.

Of note, the data of the present study, obtained on excised BK channels and BK currents of freshly isolated smooth muscle cells, are consistent with and strongly support our suggestion that the partial inhibition of the vasodilatory effect of SNP on intact rat tail arteries induced by Rp-8-Br-PET-cGMPS [4] is due to an incomplete inhibition of PKG by Rp-8-Br-PET-cGMPS at the concentrations used. Higher concentrations of Rp-8-Br-PET-cGMPS could not be employed because they might stimulate basal PKG activity [6,7]. 

In summary, the data of the present study show that the increase in the outward current of freshly isolated rat tail artery smooth muscle cells after application of SNP is due to an SNP-induced stimulation of a BK current. Further, data from excised BK channels, freshly isolated single smooth muscle cells and intact vessel preparations from rat tail artery consistently show that stimulation of the BK current after SNP application is solely due to NO released from SNP. Moreover, we did not obtain any evidence that the NO-donor-induced activation of excised BK channels observed in our study could be due to a direct action of NO on the BK channel. Thus, the stimulation of the BK current observed in our study after application of SNP is most likely mediated only by PKG. These data fill a gap, at the molecular level, in our recent extensive study [4] addressing the role of vascular smooth muscle BK channels in NO-induced vasodilation. In addition, they add to the already existing data, collected by several groups, on the mechanisms of NO-induced activation of vascular smooth muscle BK channels and, thus, contribute to a better understanding of the extent to which these mechanisms contribute to the NO-dependent regulation of BK channels.

## 4. Materials and Methods

The investigation is in accordance with the US Guide for the Care and Use of Laboratory Animals (Eighth edition, National Academy of Sciences, 2011). Approval for the use of laboratory animals in this study was granted by a governmental committee on animal welfare.

### 4.1. Cell Isolation

Cell isolation was performed using a method that has long been established in our laboratory and has been successfully used in a number of patch-clamp studies. This procedure provides single smooth muscle cells from rat tail arteries with well-preserved intracellular signaling pathways, in particular PKC- [15], PKG- [31] and PKA- [19,31,32] dependent signaling. Moreover, the activity of BK channels is long-lasting and stable, as observed in whole-cell and single-channel recordings [15,19,31,32]. Briefly, male Wistar-Kyoto rats, 16–25 weeks old, were sacrificed under CO_2_ narcosis by decapitation. The rat tail was removed and placed into a physiological salt solution containing (in mM): 145 NaCl, 4.5 KCl, 1.2 NaH_2_PO_4_, 1.0 MgSO_4_, 0.1 CaCl_2_, 0.025 EDTA, 5 HEPES at pH 7.3 at 4 °C. Tail arteries were dissected free and cleaned of connective tissue. A 5 to 10 mm-long piece was then transferred into a microtube containing 1 mL of an enzyme solution and stored there overnight at 4 °C. The enzyme solution consisted of (in mM): 110 NaCl, 5 KCl, 0.16 CaCl_2_, 2 MgCl_2_, 10 NaHEPES, 10 NaHCO_3_, 0.5 KH_2_PO_4_, 0.5 NaH_2_PO_4_, 10 glucose, 0.49 EDTA, and 10 taurine at pH 7.0, as well as 1.5 mg/mL papain, 1.6 mg/mL albumin and 0.4 mg/mL DL-dithiothreitol. The next day, the microtube with the vessel was incubated for 5–20 min at 37 °C. Single cells were released by trituration with a polyethylene pipette into the experimental bath solution. Only spindle-shaped cells with a voluminous appearance in phase contrast were used for further experiments. The experimental bath solution contained: in whole-cell experiments (in mM), 135 NaCl, 6 KCl, 1 MgCl_2_, 0.1 CaCl_2_, 3 EGTA (purity 96%) and 10 HEPES at pH 7.4 having a free calcium concentration <100 nM; and in inside-out experiments (in mM), 140 KCl, 1 MgCl_2_, 3 EGTA (purity 96%), 10 HEPES, and an appropriate amount of CaCl_2_ to obtain a free calcium concentration of 300 nM at pH 7.4. The free calcium concentrations were calculated with the following apparent reaction constants at pH 7.4: log K_CaEGTA_ = 7.17, log K_MgEGTA_ = 1.93 [33]. The pipette solution contained: in whole-cell experiments (in mM) 102 KCl, 10 NaCl, 1 MgCl_2_, 1 CaCl_2_, 0.1 MgATP, 10 EGTA (purity 96%), 10 HEPES giving a free calcium concentration <10 nM at pH 7.4; and in inside-out experiments (in mM), 130 NaCl, 15 KCl, 1 MgCl_2_, 3 EGTA (purity 96%), 10 HEPES and an appropriate amount of CaCl_2_ to obtain a free calcium concentration of 300 nM at pH 7.4. 

### 4.2. Patch-Clamp Recording

All experiments were performed at room temperature (22–24 °C). Patch pipettes were prepared from borosilicate glass (WP Instruments, Berlin, Germany) and had a resistance in the range of 2–5 MOhm. Recordings were made with an Axopatch 200 amplifier (Axon Instruments, Burlingame, CA, USA) with the whole-cell and the inside-out patch-clamp configurations. In the whole-cell experiments, stimulation of currents with pulse and ramp protocols, data sampling at a rate of 1 kHz, and data analysis were all performed with the software package ISO2 (MFK, Frankfurt, Germany). Stability of the access resistance was tested regularly during the course of the experiment. Whole-cell current amplitudes were measured during the last 200 ms of the current traces and determined after calculation of the mean of three consecutive traces. Single channel data were stored on a DTR-1800 data recorder (Biologic, Seyssinet-Pariset, France) and later replayed for analysis, where they were filtered at 1 kHz with use of an eight-pole Bessel filter (model 902, Frequency Devices, Ottawa, IL, USA) and digitized at 5 kHz. Thereafter, they were analyzed off-line with the software package ASCD (G. Droogmans, Lab. Fysiologie, KU Leuven, Belgium). The single channel amplitudes were determined by fitting Gaussian distributions to the amplitude histograms of the closed and the open state, respectively. The activity of the channel in a patch was determined as NPo, where Po is the open probability of one channel and N is the number of channels in the patch, which could not be determined in most cases because of the low level of activity. NPo was calculated as the sum of the times spent at current levels corresponding to 1, 2, …, N channels open multiplied by the number of open channels, and this sum was then divided by the total registration time. The registration time was 6–12 min. All potentials are expressed as membrane potentials.

In whole-cell investigations, experimental agents were applied to cells with use of a pipette placed at a distance from the cell and filled with the agent dissolved in the whole-cell bath solution. Application lasted 3–4 min, a period sufficient to obtain a new steady state of the whole-cell current. In the same manner, the whole-cell bath solution without agent was applied to obtain an appropriate control for the statistical evaluation of the results. In inside-out investigations, experimental agents were applied to patches by adding the agent dissolved in the inside-out bath solution directly to the bath. Thus, after addition, the agent was continuously present throughout the experiment. In preliminary experiments, other methods of agent application to inside-out patches such as bath exchange or constant superfusion had been tested by application of solutions with different calcium concentrations. It was found that, only with the direct application method, the occurrence of technical drawbacks, especially loss of patches, vesicle formation and run-down of channel activity, was sufficiently infrequent to obtain the necessary number of reproducible experiments for statistical analysis of the results. 

The pharmacological tools used—the NO-scavenger hydroxocobalamin and the PKG-inhibitor Rp-8-Br-PET-cGMPS—have been tested in pilot experiments for their interaction with NO. NO concentration was measured with a Malinski type porphyrinic NO sensor (Biologic, Seyssinet-Pariset, France). In the presence of hydroxocobalamin, NO was undetectable upon addition of NO up to a concentration of 800 nM. In the presence of Rp-8-Br-PET-cGMPS, NO concentrations tested up to 800 nM were evidently reduced by 40%. However, calibration curves of the electrode obtained in Rp-8-Br-PET-cGMPS-free solutions before and after testing Rp-8-Br-PET-cGMPS were considerably different, demonstrating an interaction of Rp-8-Br-PET-cGMPS with the electrode. Thus, a large part of the apparent reduction in NO concentration is caused by a change in electrode properties and it was concluded that there is only a small, if any, reduction in NO concentration in Rp-8-Br-PET-cGMPS-containing solutions. 

### 4.3. Drugs and Chemicals

Albumin, DL-dithiothreitol, hydroxocobalamin, MgATP, cGMP, sodium nitroprusside, and the salts for the solutions were obtained from Sigma (Deisenhofen, Germany). Papain was purchased from Ferak (Berlin, Germany). SNAP (S-nitroso-N-acetyl-penicillamine) was from Calbiochem (Bad Soden, Germany), DEA/NO from ALEXIS (Grünberg, Germany) and iberiotoxin from RBI (Cologne, Germany). Rp-8-Br-PET-cGMPS was obtained from Biolog (Bremen, Germany). The catalytic subunit of PKG was from Promega (Mannheim, Germany).

### 4.4. Statistics

All data are presented as means ± SE; n is the number of cells. Statistical analysis was performed with use of a one-sample *t*-test, independent sample *t*-test, Mann–Whitney test, or Kruskal–Wallis test with Dunn’s post hoc test, as appropriate (SPSS 9.0 for Windows, IBM, Ehningen, Germany).

## Data Availability

All data generated during this study are available from the corresponding author on reasonable request.

## References

[1] Williams D.L., Katz G.M., Roy Contancin L., Reuben J.P. (1988). Guanosine 5′-monophosphate modulates gating of high-conductance Ca^2+^-activated K^+^ channels in vascular smooth muscle cells. Proc. Natl. Acad. Sci. USA.

[2] Robertson B.E., Schubert R., Hescheler J., Nelson M.T. (1993). cGMP-Dependent Protein Kinase Activates Ca-Activated K- Channels in Cerebral Artery Smooth Muscle Cells. Am. J. Physiol.

[3] Archer S.L., Huang J.M.C., Hampl V., Nelson D.P., Shultz P.J., Weir E.K. (1994). Nitric oxide and cGMP cause vasorelaxation by activation of a charybdotoxin-sensitive K channel by cGMP-dependent protein kinase. Proc. Natl. Acad. Sci. USA.

[4] Schmid J., Muller B., Heppeler D., Gaynullina D., Kassmann M., Gagov H., Mladenov M., Gollasch M., Schubert R. (2018). The Unexpected Role of Calcium-Activated Potassium Channels: Limitation of NO-Induced Arterial Relaxation. J. Am. Heart Assoc..

[5] Butt E., Pöhler D., Genieser H.-G., Huggins J.P., Bucher B. (1995). Inhibition of cyclic GMP-dependent protein kinase-mediated effects by (Rp)-8-bromo-PET-cyclic GMPS. Br. J. Pharmacol..

[6] Taylor M.S., Okwuchukwuasanya C., Nickl C.K., Tegge W., Brayden J.E., Dostmann W.R. (2004). Inhibition of cGMP-dependent protein kinase by the cell-permeable peptide DT-2 reveals a novel mechanism of vasoregulation. Mol. Pharmacol..

[7] Valtcheva N., Nestorov P., Beck A., Russwurm M., Hillenbrand M., Weinmeister P., Feil R. (2009). The commonly used cGMP-dependent protein kinase type I (cGKI) inhibitor Rp-8-Br-PET-cGMPS can activate cGKI in vitro and in intact cells. J. Biol. Chem..

[8] Taniguchi J., Furukawa K., Shigekawa M. (1993). Maxi K^+^ Channels Are Stimulated by Cyclic Guanosine Monophosphate-Dependent Protein Kinase in Canine Coronary Artery Smooth Muscle Cells. Pflüg. Arch.-Eur. J. Physiol..

[9] Peng W., Hoidal J.R., Farrukh I.S. (1996). Regulation of Ca^2+^-activated K^+^ channels in pulmonary vascular smooth muscle cells—Role of nitric oxide. J. Appl. Physiol..

[10] Sausbier M., Schubert R., Voigt V., Hirneiss C., Pfeifer A., Korth M., Kleppisch T., Ruth P., Hofmann F. (2000). Mechanisms of NO/cGMP-dependent vasorelaxation. Circ. Res..

[11] Kyle B.D., Mishra R.C., Braun A.P. (2017). The augmentation of BK channel activity by nitric oxide signaling in rat cerebral arteries involves co-localized regulatory elements. J. Cereb. Blood Flow Metab..

[12] Fukao M., Mason H.S., Britton F.C., Kenyon J.L., Horowitz B., Keef K.D. (1999). Cyclic GMP-dependent protein kinase activates cloned BK_Ca_ channels expressed in mammalian cells by direct phosphorylation at serine 1072. J. Biol. Chem..

[13] Bolotina V.M., Najibi S., Palacino J.J., Pagano P.J., Cohen R.A. (1994). Nitric Oxide Directly Activates Calcium-Dependent Potassium Channels in Vascular Smooth Muscle. Nature.

[14] Mistry D.K., Garland C.J. (1998). Nitric oxide (NO)-induced activation of large conductance Ca^2+^-dependent K^+^ channels (BK_Ca_) in smooth muscle cells isolated from the rat mesenteric artery. Br. J. Pharmacol..

[15] Schubert R., Noack T., Serebryakov V.N. (1999). Protein kinase C reduces the K_Ca_ current of rat tail artery smooth muscle cells. Am. J. Physiol..

[16] Galvez A., Gimenez Gallego G., Reuben J.P., Roy Contancin L., Feigenbaum P., Kaczorowski G.J., Garcia M.L. (1990). Purification and characterization of a unique, potent, peptidyl probe for the high conductance calcium-activated potassium channel from venom of the scorpion Buthus tamulus. J. Biol. Chem..

[17] Giangiacomo K.M., Garcia M.L., McManus O.B. (1992). Mechanism of Iberiotoxin Block of the Large-Conductance Calcium-Activated Potassium Channel from Bovine Aortic Smooth Muscle. Biochemistry.

[18] Kruszyna H., Magyar J.S., Rochelle L.G., Russell M.A., Smith R.P., Wilcox D.E. (1998). Spectroscopic studies of nitric oxide (NO) interactions with cobalamins: Reaction of NO with superoxocobalamin(III) likely accounts for cobalamin reversal of the biological effects of NO. J. Pharmacol. Exp. Ther..

[19] Schubert R., Serebryakov V.N., Engel H., Hopp H.-H. (1996). Iloprost activates K_Ca_ channels of vascular smooth muscle cells: Role of cAMP-dependent protein kinase. Am. J. Physiol..

[20] Bates J.N., Baker M.T., Guerra R., Harrison D.G. (1991). Nitric oxide generation from nitroprusside by vascular tissue. Evidence that reduction of the nitroprusside anion and cyanide loss are required. Biochem. Pharmacol..

[21] Filipovic M.R., Eberhardt M., Prokopovic V., Mijuskovic A., Orescanin-Dusic Z., Reeh P., Ivanovic-Burmazovic I. (2013). Beyond H_2_S and NO interplay: Hydrogen sulfide and nitroprusside react directly to give nitroxyl (HNO). A new pharmacological source of HNO. J. Med. Chem..

[22] Zhao Y., Vanhoutte P.M., Leung S.W. (2015). Vascular nitric oxide: Beyond eNOS. J. Pharmacol. Sci..

[23] Francis S.H., Busch J.L., Corbin J.D., Sibley D. (2010). cGMP-dependent protein kinases and cGMP phosphodiesterases in nitric oxide and cGMP action. Pharmacol. Rev..

[24] Mughal A., Sun C., O’Rourke S.T. (2018). Activation of Large Conductance, Calcium-Activated Potassium Channels by Nitric Oxide Mediates Apelin-Induced Relaxation of Isolated Rat Coronary Arteries. J. Pharmacol. Exp. Ther..

[25] Carrier G.O., Fuchs L.C., Winecoff A.P., Giulumian A.D., White R.E. (1997). Nitrovasodilators relax mesenteric microvessels by cGMP-induced stimulation of Ca-activated K channels. Am. J. Physiol. Heart Circ. Physiol..

[26] McCobb D.P., Fowler N.L., Featherstone T., Lingle C.J., Saito M., Krause J.E., Salkoff L. (1995). A human calcium-activated potassium channel gene expressed in vascular smooth muscle. Am. J. Physiol Heart. Circ. Physiol..

[27] Schubert R., Nelson M.T. (2001). Protein kinases: Tuners of the BK_Ca_ channel in smooth muscle. Trends Pharmacol. Sci..

[28] Silberberg S.D., Lagrutta A., Adelman J.P., Magleby K.L. (1996). Wanderlust kinetics and variable Ca^2+^-sensitivity of dslo, a large conductance Ca^2+^-activated K^+^ channel, expressed in oocytes. Biophys. J..

[29] Miyoshi H., Nakaya Y., Moritoki H. (1994). Nonendothelial-derived nitric oxide activates the ATP-sensitive K^+^ channel of vascular smooth muscle cells. FEBS Lett..

[30] George M.J., Shibata E.F. (1995). Regulation of calcium-activated potassium channels by S-nitrosothiol compounds and cyclic guanosine monophosphate in rabbit coronary artery myocytes. J. Investig. Med..

[31] Schubert R., Lehmann G., Serebryakov V.N., Mewes H., Hopp H.-H. (1999). cAMP-dependent protein kinase is in an active state in rat small arteries possessing a myogenic tone. Am. J. Physiol..

[32] Schubert R., Serebryakov V.N., Mewes H., Hopp H.-H. (1997). Iloprost dilates rat small arteries: Role of K_ATP_- and K_Ca_-channel activation by cAMP-dependent protein kinase. Am. J. Physiol..

[33] Schubert R. (1996). Multiple ligand-ion solutions: A guide for solution preparation and computer program understanding. J. Vasc. Res..

